# Myotonia Congenita-Associated Mutations in Chloride Channel-1 Affect Zebrafish Body Wave Swimming Kinematics

**DOI:** 10.1371/journal.pone.0103445

**Published:** 2014-08-01

**Authors:** Wei Cheng, Jing Tian, Jean-Marc Burgunder, Walter Hunziker, How-Lung Eng

**Affiliations:** 1 Epithelial Cell Biology Laboratory, Institute of Molecular and Cell Biology, Agency for Science Technology and Research, Singapore, Singapore; 2 Institute for Infocomm Research, Agency for Science Technology and Research, Singapore, Singapore; 3 Department of Neurology and Deparment of Clinical Research, University of Bern, Bern, Switzerland; 4 Department of Physiology, National University of Singapore, Singapore, Singapore; 5 Singapore Eye Research Institute, Singapore, Singapore; University of Florida, United States of America

## Abstract

Myotonia congenita is a human muscle disorder caused by mutations in *CLCN1*, which encodes human chloride channel 1 (CLCN1). Zebrafish is becoming an increasingly useful model for human diseases, including muscle disorders. In this study, we generated transgenic zebrafish expressing, under the control of a muscle specific promoter, human CLCN1 carrying mutations that have been identified in human patients suffering from myotonia congenita. We developed video analytic tools that are able to provide precise quantitative measurements of movement abnormalities in order to analyse the effect of these CLCN1 mutations on adult transgenic zebrafish swimming. Two new parameters for body-wave kinematics of swimming reveal changes in body curvature and tail offset in transgenic zebrafish expressing the disease-associated CLCN1 mutants, presumably due to their effect on muscle function. The capability of the developed video analytic tool to distinguish wild-type from transgenic zebrafish could provide a useful asset to screen for compounds that reverse the disease phenotype, and may be applicable to other movement disorders besides myotonia congenita.

## Introduction

Myotonia congenita (MC) is an inherited muscle disorder that affects muscle relaxation. It is characterized by muscle stiffness after a voluntary contraction, which typically decreases with repetitive movement [1]. Human myotonia congenita can be inherited in an autosomal recessive (Becker type) or autosomal dominant (Thomsen type) [2]. The clinical evaluations of the myotonia congenita include the expression of muscle stiffness or muscle weakness, muscle hypertrophy, percussion and electromyographic myotonia.

CLCN1, a type 1 chloride channel which is expressed almost exclusively in skeletal muscle fibers, plays important roles for the physiological functions of skeletal muscles [3], [4]. CLCN1 is a voltage-dependent ion channel, which is activated by depolarization. Sequence analysis of *CLCN1* showed that mutations linked to myotonia are scattered over the entire sequence of the channel protein, and include insertion/deletions, missense, nonsense, and splicing mutations [5]. *CLCN1* mutations impair CLCN1 functions and render the plasma membrane hyper-excitable, leading to clinical myotonia and typical electromyographic changes [1]. Two novel CLCN1 mutations have been found in Chinese and the physiological effects of the mutations were examined by expression of the channels in *Xenopus* oocytes. The results demonstrated that both mutations shifted the voltage required for half-maximal activation. The larger effect was seen in the compound heterozygous situation combining the I553F and the H555N mutations [6].

Zebrafish (*Danio rerio*) has become a widely used model system for the study of development and gene function. Zebrafish are vertebrates with many physiological similarities to humans [7]. They are relatively small fish and allow easy handling and maintenance at low cost [8]. In addition, zebrafish behavior can be easily observed and analyzed in a controlled environment [9]. The availability of efficient techniques for manipulation of gene expression has made the zebrafish an attractive and efficient system to study human diseases including movement disorders and myopathies [10]–[12]. In this study, we generated zebrafish models for *myotonia congenita* by expressing human CLCN1 (hCLCN1) carrying mutations associated with human patients suffering from myotonia under the control of the muscle specific promoter of the α-actin gene. These transgenic fish were used to develop novel video analytic tools for zebrafish tracking, feature extraction and movement analysis, with a particular focus on individual fish movement parameters.

A video analytic tool should be able to provide precise quantitative measurements of zebrafish behavioral abnormalities, which can be used for screening of compounds that induce a change from normal or, in the case of a disease model, from abnormal movement. Such analyses benefit greatly from computer vision techniques, which can accurately and efficiently monitor complex locomotor characteristics [13]. Until recently, the quantification of zebrafish behavior was performed manually, making it vulnerable to human error and incorrect data interpretation, thereby reducing the validity of an experiment. While visual monitoring of behavior is time-consuming and prone to subjective variation, the development of dedicated computer vision techniques is desired in exploiting the information contained in the acquired image and video data [14], [15]. Computerized video analytic tools that analyze zebrafish movements provide standardized observation of behavioral measurements and reduce human errors. Video analytic technology helps fast and objective quantification of zebrafish behavior [16]–[18]. These tools also provide basic measurements (e.g., distance traveled, velocity) which cannot be scored manually [19]. However, these systems analyze the fish only as a point and cannot quantify body wave kinematics of swimming. Several studies have been developed to examine details of zebrafish body waving in video recorded with high frame rates. However, a majority of these studies have focused on larval locomotion [20]–[24], less is known for adult zebrafish, where the escape response of wild-type zebrafish and transgenic zebrafish have been studied [25].

In this study, we present a video analytic tool that is able to provide precise quantitative measurements of behavioral abnormalities for detecting effects on muscular or nervous system function. The tool analyses the movement behavior of a single adult Zebrafish in an automated and batch manner. Two new body-waving parameters are presented that expand the currently available toolbox of zebrafish motion measurements. We demonstrate the capability of the developed video analytic tool to distinguish wild-type zebrafish from transgenic lines that express disease-associated mutations in CLCN1. The mutant CLCN1 channels affect zebrafish body curvature and tail offset as a result from effects on muscle function. The zebrafish model could provide additional insights into myotonia congenita pathogenesis and, combined with the video analytic tools, be used for automated small molecule screening and monitoring of disease progression.

## Materials and Methods

### Construction of α-actin:hCLCN1-EGFP and α-actin:hCLCN1-IRES-EGFP plasmids

A 3.9 kb α-actin promoter was amplified using PCR from α-actin-EGFP construct [26]. N-terminus Flag-tagged human wild type (WT) or mutated chloride channel gene 1 (hCLCN1) were amplified using PCR [6] and fused with EGFP or IRES-EGFP (Clontech, Mountain View, CA, USA) followed by an SV40 poly(A) signal. These fragments were placed downstream of the α-actin promoter. The α-actin promoter controlled hCLCN1-EGFP or hCLCN1-IRES-EGFP was inserted into the mini Tol2 vector for generating the transgenic zebrafish. All DNA constructs were verified by sequencing using ABI automated DNA sequencing system.

### Construct DNA preparation and microinjection into zebrafish embryos

Plasmid DNA was prepared using the Qiagen miniprep kit. pT3Ts-Tol2 plasmid was linearized with SmaI and used as a template for Tol2 transposase mRNA synthesis *in* *vitro*. Tol2 transposase mRNA was synthesized using the mMessage Machine T3 kit from Ambion according to the manufacturer’s manual. One-cell stage WT embryos were co-injected with 25 ng/µl of the DNA construct and 25 ng/µl of the mRNA. After 24 hours embryos were sorted under the fluorescence microscope. Surviving fish positive for Green fluorescent protein (EGFP) expression were raised to sexual maturity and crossed in pairs to identify germline chimeras. All animal experimentation was approved by the Institutional Animal Care and Use Committee of the Biomedical Research Council of the Agency for Science, Technology and Research, Singapore (IACUC protocol #120717).

### Identification of transgenic zebrafish

Adult fish were crossed in pairs and the embryos were observed under the fluorescent microscope to screen for EGFP expression. Fish that transmitted the transgene to the next generation were then outcrossed with wild-type fish. EGFP expression in the offsprings was analyzed to identify which one of each pair was the germline chimera (F0). F1 progeny of each F0 fish were then raised to adulthood. Transgenic F1 fish were identified by fluorescence microscopy of F2 embryos after outcross to wild type fish. 3 dpf live F1 embryos were observed under the fluorescent microscope equipped with an Olympus DP70 digital camera. Pictures were taken using the same settings. The GFP fluorescence intensity was quantitated using the Image J software.

### Genomic DNA preparation and PCR screening

Zebrafish embryos were incubated with 30 µl of TE buffer and protease K at 55°C over night. The sample was then heated to 95°C for 10 min to inactivate proteinase K, 1 µl of the lysate was used for PCR using the primer specific to hCLCN1 and the EGFP genes.

### Protein extraction and Western blot analysis

2 dpf embryos were dechorionated with 1 mg/ml pronase and washed 2–3 times in ice cold PBS supplemented with 1x cocktail protease inhibitors (Roche). The embryos were de-yolked by pipetting up and down with yellow tips followed by centrifugation at 4°C, 200×g for 5 min. The pellet was lysed in lysis buffer (63 mM Tris-HCl pH 6.8, 10% glycerol, 5% β-mercaptoethanol, 3.5% sodium dodecyl sulphate) at 2 µl per embryo, homogenized with a 26G needle and boiled for 5 min. After centrifugation in a microcentrifuge for 5 minutes, the supernatant was collected and analyzed by SDS-PAGE and Western blot using rabbit anti-Flag and suitable secondary antibodies (Sigma).

### Video recording setup

In our experiments, we have examined two datasets of zebrafish. The first dataset consists of four types of zebrafish: wild-type, hCLCN1-EGFP, hCLCN1^L844F^-EGFP, hCLCN1^I553F/H555N^-EGFP. The second dataset consists of four types of zebrafish: wild-type, hCLCN1-IRES-EGFP, hCLCN1^L844F^-IRES-EGFP, hCLCN1^I553F/H555N^-IRES-EGFP. The first dataset contains of 24 zebrafish, while the second dataset contains of 20 zebrafish. The AOS™ SPRI camera is mounted above the fish tank and the video is recorded from the top view. The video is recorded with a frame rate 250 frames per second, and 480×640 pixels per frame. Each video contains 4000 frames (i.e., 16 seconds). The recorded video is stored in AVI format without applying video compression technologies. Representative videos of the seven different zebrafish lines are included as supporting files (Video S1–S8).

### Video analytic tools

The developed video analytic system is able to perform continuous and automated zebrafish monitoring, by tracking, extracting features and analyzing zebrafish swimming behavior. We record the video of zebrafish swimming using a camera that is mounted above the fish tank. The video is recorded from the top view. With this image acquisition setup, it is possible to examine fish body swimming kinematics, which is critical to serve as foundation for differentiating normal and abnormal zebrafish. We used Matlab programming language to develop our own video analytic algorithms as follows.

First, the moving zebrafish was detected and tracked in the video using background subtraction and object tracking [27], which was developed using Matlab programming language with its built-in image processing toolbox. First, a median frame of the video is chosen as the background frame, where the intensity value of each pixel of this background frame is calculated as the median value of all intensities values of same pixel location of all frames. Next, the fish body is tracked as follows. The fish object is detected by subtracting each frame with the background frame. The largest connected component in this difference image is selected as the fish body. Then, the boundary of the fish body is extracted, followed by various quantitative measurements that are obtained as follows. Among various developed motion representations in the literature, the silhouettes-based features are desirable for motion representation since it is invariant to luminance, color and texture of the moving objects as well as the background. By tracking the fish, we were able to provide each zebrafish a unique label that enables individual tracking, and create temporal profile for analysis of individual motion activity. We further manually split the video sequence into smaller video clips containing one full body waving cycle. A body waving cycle is defined as a video segment containing one body waving zebrafish, where the fish starts from the straight posture and then bends its body and finally returns to straight posture.

We then extracted quantitative measurements of body waving behavior of zebrafish in a single body waving cycle (i.e., video clip). Given the segmented and tracked fish in each video frame using the aforementioned method, we extracted the skeleton of the fish and establish a geometrical model with twenty control points to describe the posture of fish [27]. These control points are uniformly distributed along the fish body from the head to the tail. We defined the following two quantitative measurements of body waving behavior of zebrafish movements.

**Figure 1:**
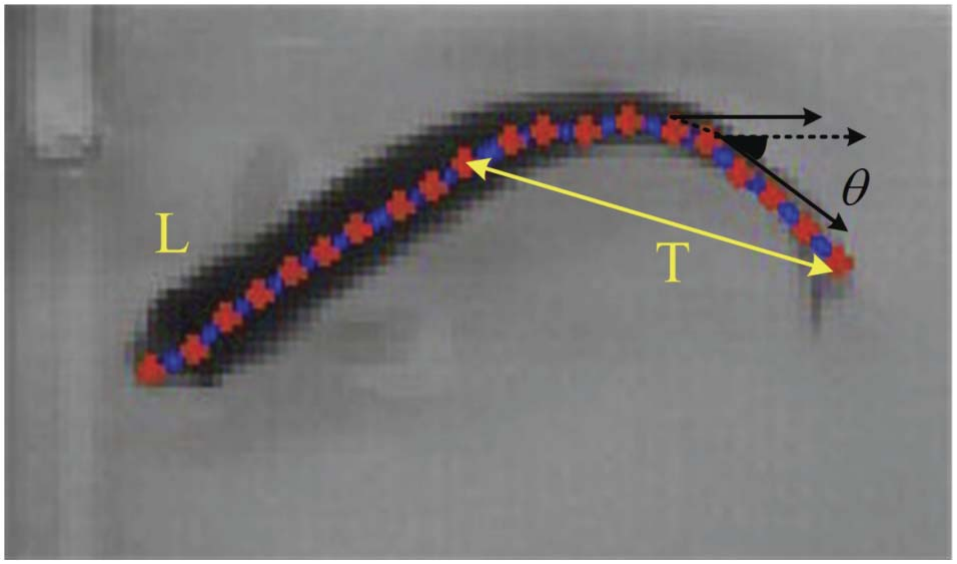
Illustration of quantitative measurements of zebrafish body waving. The fish body (blue curve) is characterized at twenty body locations (red dot). *L* is the body length, *T* is the distance between the tail and the center of fish body, 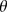 is the angular change between two consecutive body locations. Given the coordinates of two consecutive body locations [*x*(*i*), *y*(*i*)] and [*x*(*i+1*), *y*(*i+1*)], the angular change is defined as 
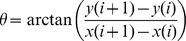
.

We extracted the curvature of fish body bends at each body location. Given an extracted fish centerline that has a set of twenty body locations, whose coordinates at the *i*-th body location is denoted as 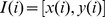, where *x*(*i*), *y*(*i*) denote the coordinates at horizontal and vertical directions, respectively. Its body curvature (denoted as *K*(*i*)) at the body location *i* can be calculated as
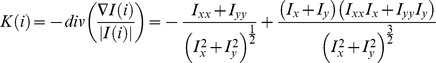
Where 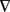 represents the gradient operator and div represents the divergence operator, four differential operators 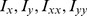 are calculated as 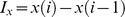, 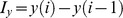, 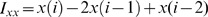, 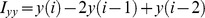, respectively. As seen in above equation, a larger body curvature value indicates larger bending of fish body, which means the larger angular change (i.e., 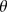 in Figure 1) along two neighboring control points. Given the coordinates of two consecutive body locations [*x*(*i*), *y*(*i*)] and [*x*(*i+1*), *y*(*i+1*)], the angular change is defined as 
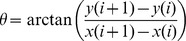
.

The second feature is to measure the tail offset of fish body waving. It is defined as the ratio between the distance of the center and tail (defined as 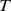 in Figure 1) and the length of whole fish body (defined as 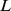 in Figure 1). A smaller tail offset value indicates larger bending of fish body. This approach does not rely on determining the tail’s displacement from a mean path of motion, and is therefore invariant to the spatial trajectory of the fish.

In addition to the aforementioned two features representing the body waving behavior of zebrafish, we defined a third feature, *travel distance*, which characterizes the motion behavior of zebrafish. It is defined as the absolute distance traveled by the fish body centroid over the period (i.e., frames) for each body waving cycle (i.e., video clip). A demonstration video and the source code of the implementation of the vidoanalytics system in Matlab are provided as supporting files Video S8 and Information S1, respectively.

**Figure 2:**
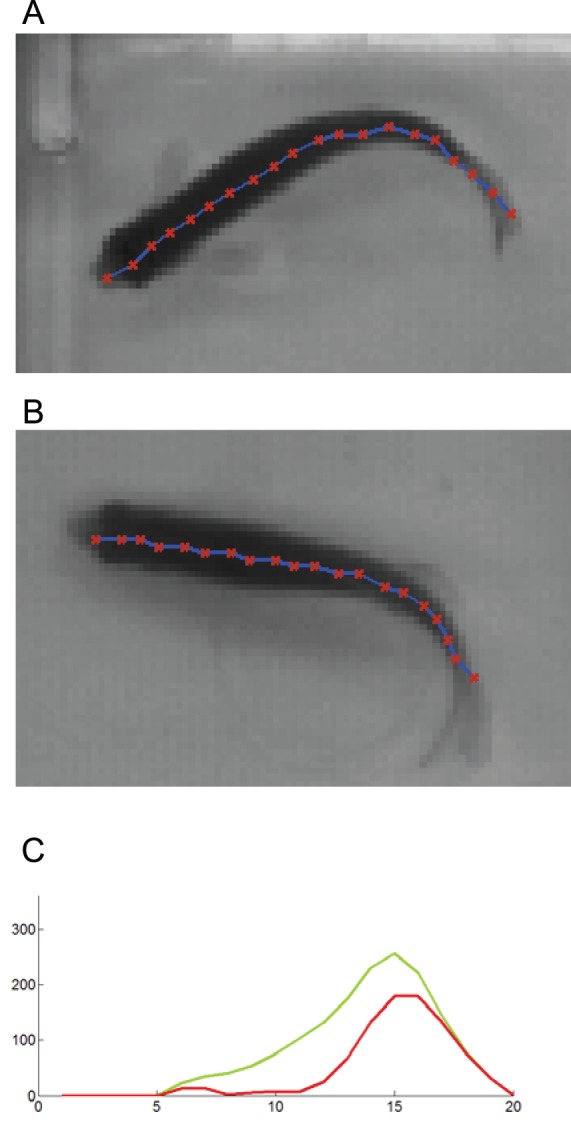
The body curvature comparison between wild-type zebrafish and transgenic zebrafish. (A) wild-type zebrafish; (B) transgenic zebrafish; (C) the comparison of curvatures measured along twenty body locations of wild-type zebrafish (green plot) and transgenic zebrafish (red plot), respectively. The fish body (blur curve) is characterized at twenty body locations (red dot) in (A) and (B). In (c), the x-axis represents the fish body location index, and the y-axis represents the body curvature value (in the unit of degree). Note that we extracted the skeleton of the fish body and used twenty control points to describe the posture of fish. These control points are uniformly distributed along the fish body skeleton from the head to the tail. The first point is located at fish head and the last point is located at fish tail. The body curvature is measured at each point location.

We compared the body curvature measurements of two different zebrafishes with different postures shown in Figures 2A–2B, respectively. For each of them, we calculated the body curvature at each body location. We further applied spatial smoothing on the calculated body curvature values using a low-pass filter. The cut-off frequency of the filter is chosen based on visual inspection of the magnitude response of the body curvature’s Fourier transform. The larger body curvature values indicate larger body bending (Figure 2C).

### Analysis of zebrafish cohorts

The recorded zebrafish movement video clip was manually split into smaller clips containing one full body waving cycle. For each body waving cycle (e.g. video clip), the fish body was tracked and the bending degree determined using body curvature and tail offset as criteria. First, we extracted the body curvature measurement of zebrafish at each frame, and then consolidated them into a matrix. Each column represents the body curvature measurements in one frame. The number of columns is equal to the number of frames of one body waving cycle. The body curvature measurement at each control point and formulate a representation of the obtained curvature measurements, *C*, where *C* is a 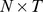 matrix with *N* being the number of measurements in one frames, and *T* the number of frames being considered as one body waving cycle of the fish. We further applied spatial smoothing and temporal smoothing on the extracted body curvature data. The spatial smoothing is applied on the body curvatures along the zebrafish body (i.e., each column) using a low-pass filter. The temporal smoothing is applied on the body curvatures at the same location of the zebrafish body (i.e., each row) using a low-pass filter. Second, we extracted the tail offset of zebrafish at each frame, which is further smoothed using a low-pass filter. The cut-off frequency of the filter is chosen based on visual inspection of the magnitude response of the body curvature and tail offset’s Fourier transform, respectively. Finally, the spatial-temporal body curvature profiles and temporal tail offset profiles were averaged over all body waving cycles of all zebrafish within same category to obtain an average body curvature profile and average tail offset profiles.

### Statistical analysis

The experimental data are analyzed using student-t test; the significance was set at p<0.05 in all experiments of this study. In each dataset, the experimental data are compared with respect to that of wild-type zebrafish. We use the star symbol to indicate the significant data in our figures.

## Results and Discussion

### Generation of stable transgenic zebrafish with muscle-specific expression of α-actin:hCLCN1-EGFP and α-actin:hCLCN1-IRES-EGFP

**Figure 3:**
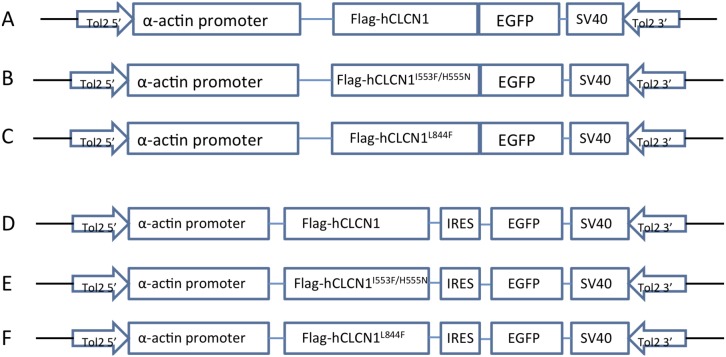
Maps of α-actin:hCLCN1-EGFP and α-actin:hCLCN1-IRES-EGFP constructs used in this study. (A–C) Three constructs expressing hCLCN1, hCLCN1^I553F/H555N^, and hCLCN1^L844F^ –green fluorescent protein (EGFP) fusions under the control of the α-actin promoter were used to produce stable transgenic zebrafish lines. EGFP was fused to the C-terminus of the flag-tagged hCLCN1. The Tol2 vector sequences are shown as thick black lines. (D-E) Three constructs expressing hCLCN1, hCLCN1^I553F/H555N^, hCLCN1^L844F^ under the control of the α-actin promoter were used to produce stable transgenic zebrafish lines. An internal ribosome entry site (IRES) element is inserted in front of EGFP to allow simultaneous expression of hCLCN1 and EGFP protein separately but from the same RNA transcript. The Tol2 vector sequence is shown as thick black lines.

Constructs for human type 1 chloride channel (hCLCN1) or mutants linked to patients suffering from myotonia congenita (MC; hCLCN1^I553F/H555N^, hCLCN1^L844F^) under the controls of the muscle specific α-actin promoter (Figure 3) were used to produce stable transgenic zebrafish lines. In one set of constructs, EGFP was clones in frame downstream of wild-type or mutated hCLCN1 to express a hCLCN1-GFP fusion protein (Figure 3A–C). In a second set, wild-type or mutated hCLCN1 and EGFP were separated by an internal ribosomal entry site (IRES; Figure 3D–F), allowing the independent expression of hCLCN1 and EGFP from the α-actin promoter.

**Figure 4:**
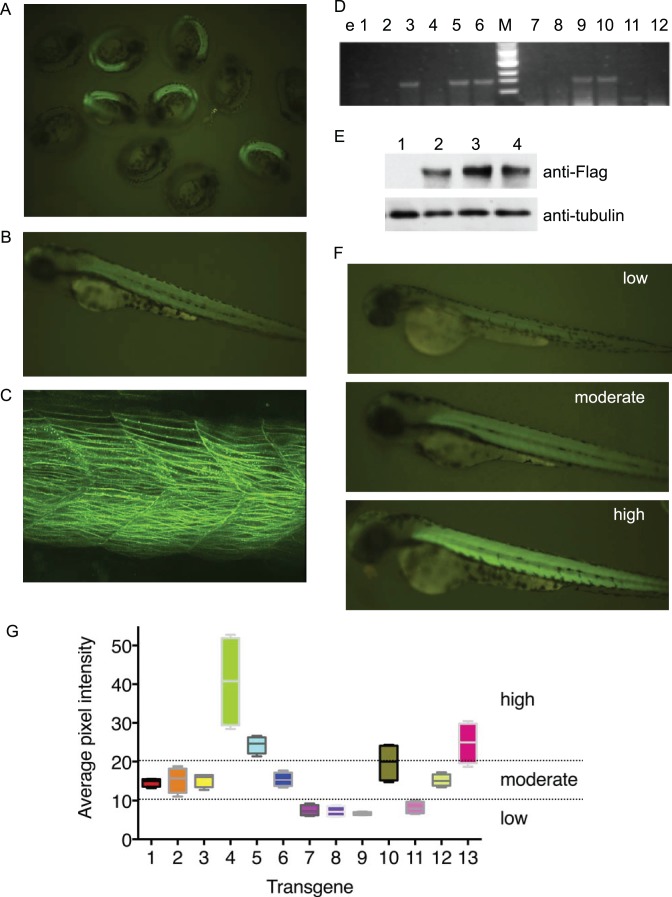
Characterization of stable transgenic zebrafish embryos using Tg (α-actin:hCLCN1^I553F/H555N^–EGFP). (A) A pool of 28 h dpf embryos from the stable transgenic founder fish showing the EGFP expression. Nonfluorescent embryos are nontransgenic siblings. (B) Embryos with positive EGFP expression show the muscle-specific expression in their trunk region. (C) Confocal microscopic image of the trunk of an F1 generation Tg (*α-actin*:hCLCN1^I553F/H555N^–EGFP) embryos at 3 dpf. EGFP expression was observed within the zebrafish muscle fibers. (D) Transgenic founders were confirmed by transmission of the CLCN1 transgene to F1 progeny by PCR. Transgenic founders were crossed with WT zebrafish, individual F1 embryos were lysed and PCR were performed using hCLCN1 specific primers and the PCR product analyzed by agarose gel electorphorsis. EGFP-positive embryos are identified based on the presence of the 1.5 kb band (e3, 5, 6, 9 and 10), while the EGFP-negative embryos lack this band (e1, 2, 4, 7, 8, 11 and 12). e; embryo. (E) Transgenic founders were confirmed by Western blot. Protein was extracted from F1 embryos at 2 dpf. Lane 1: WT AB Zebrafish, Lane 2–4: Individual stable transgenic fish expressing the 130 kDa Flag-tagged hCLCN1^I553F/H555N^–EGFP (see Materials and Methods). Actin was used as a loading control. (F) Transgenic zebrafish embryos at 3 dpf showing low, moderate or high expression of hCLCN1^I553F/H555N^–EGFP based on GFP intensity. (G) Quantification of hCLCN1^I553F/H555N^–EGFP expression and classification. For each transgene, 3 siblings of 3 dpf embryos were analyzed. Average pixel intensities (api) for EGFP fluorescence were determined on 5 areas of the trunk and the average for each sibling is plotted. Expression levels of the transgene were arbitrarily classified into low (<10 api), moderate (10–20 api) or high (>20 api). Similar results were obtained for the other transgenes (data not shown).

Each individual construct was co-injected with Tol2 transposase mRNA into one-cell stage embryos, the embryos were raised and viewed under the fluorescence microscopy after 24 hdpf. The embryos with positive EGFP expression showed an identical spatial expression pattern (Figure 4A–C) consistent with muscle specific expression of the hCLCN1-EGFP constructs by the α-actin promoter. Embryos with positive EGFP expression were selected and raised to sexual maturity, screened for germline-transmission and selected as founders (F0). For each hCLCN1 construct, three independent founders showing similar EGFP expression levels were selected for further analysis.

For each founder fish, genomic DNA from the offspring was extracted and analysed for the presence of the transgene by PCR using primers specific for hCLCN1 and EGFP. Only EGFP positive embryos showed the presence of the transgene (Figure 4D). The Flag-tagged human CLCN1 protein expression was also confirmed in transgenic zebrafish by Western Blot using an antibody against FLAG-tag (Figure 4E). These results thus demonstrated muscle specific expression of the hCLCN1 gene and its transmission from founder fish to the F1 generation.

In contrast to the similar tissue-specific expression pattern of the transgenes, EGFP expression levels as assessed by EGFP fluorescence intensities varied among the F1 generation (Figure 4F). EGFP fluorescence intensity was quantitated and the F1 transgenic fish were arbitrarily classified into three groups based on fluorescence intensity, reflecting EGFP expression levels (Figure 4G). An assessment of the swimming behavior among these different groups revealed that only transgenic fish harboring a mutant hCLCN1 construct with medium or strong expression presented with a phenotype. This probably reflects the presence of endogenous zebrafish clcn1, which may be able to compensate the effect of low amounts of the human mutant protein. Therefore, transgenic zebrafish with medium or strong EGFP fluorescence were selected for subsequent studies.

### Disease-associated mutations in CLCN1 affect zebrafish movement

**Figure 5:**
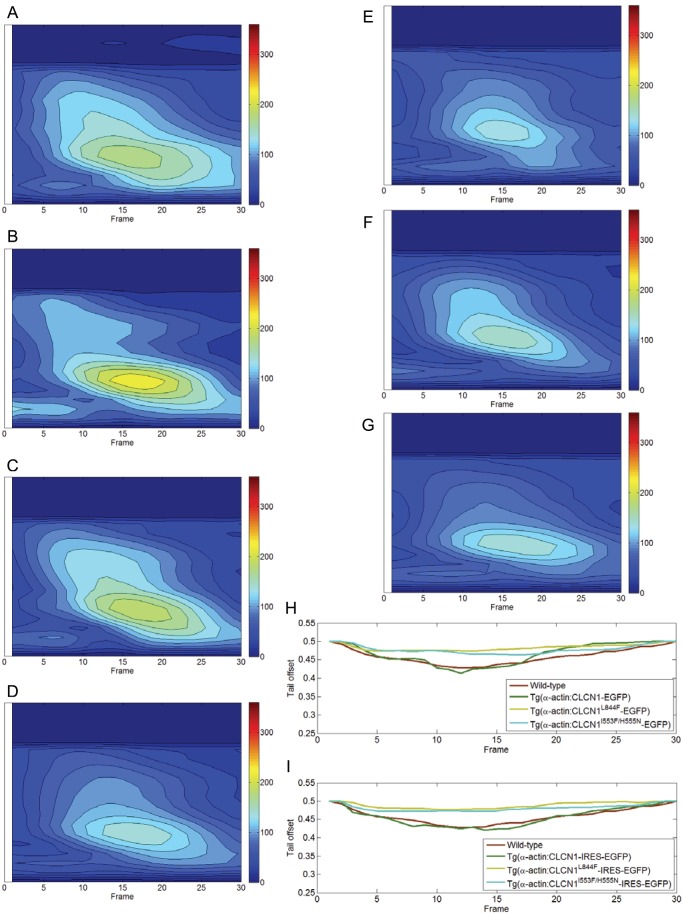
The comparison of averaging spatial-temporal body curvature profiles and averaging temporal tail offset profiles. For each zebrafish, 16-second video sequences at high frame rates were recorded. The video clip was manually split into smaller clips containing one full body waving cycle. The fish body was tracked and the bending degree determined using body curvature and tail offset as criteria. (A–G) averaging spatial-temporal body curvature profiles that are obtained from the following zebrafish: wild-type, hCLCN1-EGFP, hCLCN1^L844F^-EGFP, hCLCN1^I553F/H555N^-EGFP, hCLCN1-IRES-EGFP, hCLCN1^L844F^-IRES-EGFP, hCLCN1^I553F/H555N^-IRES-EGFP, respectively. We consolidated body curvature measurements calculated at each frame into a matrix. Each column represents the body curvature measurements in one frame. The number of columns is equal to the number of frame of one body waving cycle. We further averaged matrices over all body waving cycles of all zebrafish within same category to obtain an averaging body curvature profile. Red color indicates higher curvature value and larger bending of fish body. The Y-axis represents the body curvature measurements in one frame. The X-axis represents frame index of one video segment of one body waving cycle. (H–I) Consolidated tail offset measurements calculated at each frame into a vector. The vectors are averaged over all body waving cycles of all zebrafish within same category to obtain an averaging tail offset profile. Larger tail offset value indicates smaller bending of fish body.

To determine if zebrafish expressing mutant human CLCN1 showed differences in body bending during swimming, we used high frame video recording to track the fish body and measure its bending degree using body curvature and tail offset as parameters. The transgenic lines expressing the disease-associated CLCN1 mutants showed a smaller angular change along their body as compared to control zebrafish or fish expressing wild-type hCLCN1 (Figures 5A–5G). A smaller body curvature value means a smaller angular change along two neighboring control points along the fish body, and consequently indicates smaller bending of the fish body. On the other hand, the transgenic lines expressing the disease-associated CLCN1 mutants presented larger tail offset values (Figures 5H–5I). A larger tail offset value indicates that the fish tail is far from the centroid of fish body, and consequently the fish body has a smaller bending. The above observations are expected if the transgenic zebrafish have a defect in muscle function as a result from the mutations in *CLCN1*. To obtain such ‘averaging’ profile, we need to use same number (i.e., 30 frames in our studies) of frames in body waving cycle. First, if the body waving cycle has more than 30 frames, then we uniformly selected 30 frames from this body waving cycle. In this case, these 30 frames are not continuous. Second, if the body waving cycle has less than 30 frames, we selected a few more frames before/after this body waving cycle. In this case, these 30 frames are still continuous.

**Figure 6:**
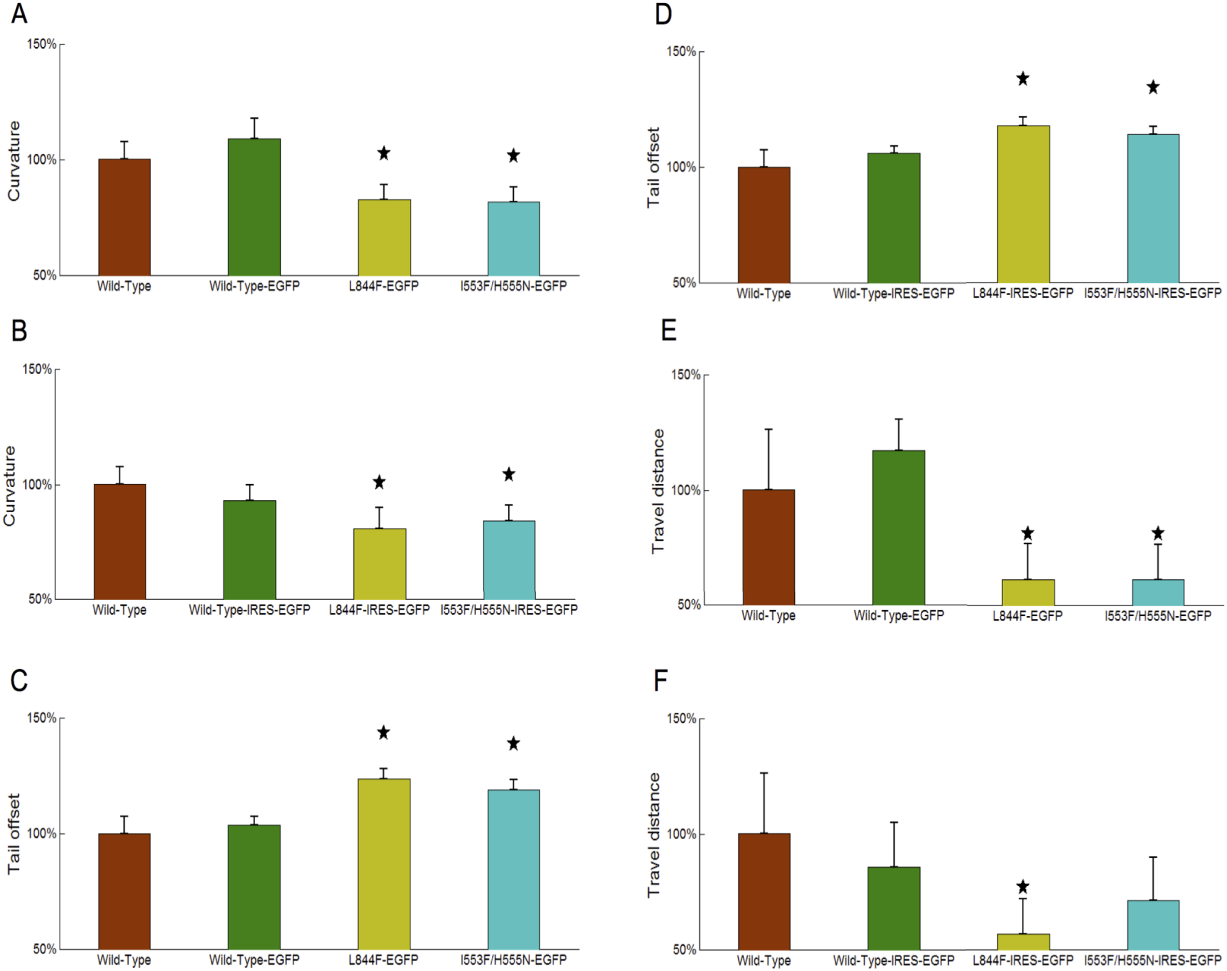
Comparison of body curvature, tail offset and swimming distance measurements between wild-type, control and transgenic zebrafish. We selected the largest body curvature value (A–B); smallest tail offset value (C–D), and travel distance (E–F) from each body waving cycle. All measurements are first averaged over all video clips within same category. The averaged measurements are further divided by the mean measurement of wild-type zebrafish. The measurements are plotted as mean +/− standard deviation. The star symbol in the figure indicates a significant difference (p<0.05) in the Student-t test.

We studied body bending and swimming capability of different categories of zebrafish. We selected the largest body curvature value; smallest tail offset value, and travel distance from each body waving cycle. These measurements are averaged over all video clips within the same category and compared in Figure 6. Transgenic zebrafish expressing the disease-associated CLCN1 mutants show smaller body curvatures (Figures 6A and 6B) and larger tail offsets (Figures 6C and 6D) than those of wild-type or control zebrafish, which is expected to result in the observed shorter distances travelled by the transgenic zebrafish expressing the disease-associated CLCN1 mutants (Figures 6E and 6F).

### Correlation with clinical manifestations in myotonia congentita patients

CLCN1 mutations under study, L884F and the combined I553F with H555N, lead to typical autosomal dominantly inherited phenotypes in human [6]. The L884F mutation leads to protein sequence variation in the cystathionin binding domain and the I553F/H555N to a change in the extracellular loop between two transmembrane helices. The two mutations shift the voltage required for activation [6], the first more than the second, which is compatible with the slightly more severe clinical phenotype. Our results in zebrafish are compatible with these clinical and physiological features. A more generalised comparison between genotype-phenotype in human and zebrafish will need a larger cohort of zebrafish examining more mutations in other channel domains. Our observation in zebrafish of a smaller body curvature and larger tail offset in mutated *CLCN1* transgenic animals is compatible with an increased muscle stiffness, leading to dicreased motor function as measured by decreased travel distance. Muscle stiffness and decrased speed of motion are the typical symptoms presented by patients with myotonia.

### Conclusion

The present study utilizes a novel video analytic approach to analyze adult zebrafish movement behavior using high frame rate recording. The proposed video analytic tool is able to examine body bending behavior and motion behavior of zebrafish. Body bending is monitored using body curvature and tail offset as parameters. Motion behavior is characterized distance travelled per swimming cycle. The mutated *CLCN1* transgenic zebrafish are affected in their ability to bend their body during movement, resulting in weaker force generation during a swimming cycle and thus shorter distance travelled. Our study show that integrated assessment of body bending and movement behavioral parameters can be used to characterize modest impairment of muscle function in zebrafish that reflect observations made in the clinic on patients with myotonia congenita.

## Supporting Information

Video S1 **Video file showing a movie of wild-type zebrafish.** The video is recorded using the AOS™ SPRI camera that is mounted above the fish tank with a frame rate 250 frames per second.
(AVI)

Video S2 **Video file showing a movie of hCLCN1-EGFP zebrafish.** The video is recorded using the AOS™ SPRI camera that is mounted above the fish tank with a frame rate 250 frames per second.
(AVI)

Video S3 **Video file showing a movie of hCLCN1^L844F^-EGFP zebrafish.** The video is recorded using the AOS™ SPRI camera that is mounted above the fish tank with a frame rate 250 frames per second.
(AVI)

Video S4 **Video file showing a movie of hCLCN1^I553F/H555N^-EGFP zebrafish.** The video is recorded using the AOS™ SPRI camera that is mounted above the fish tank with a frame rate 250 frames per second.
(AVI)

Video S5 **Video file showing a movie of hCLCN1-IRES-EGFP zebrafish.** The video is recorded using the AOS™ SPRI camera that is mounted above the fish tank with a frame rate 250 frames per second.
(AVI)

Video S6 **Video file showing a movie of hCLCN1^L844F^-IRES-EGFP zebrafish.** The video is recorded using the AOS™ SPRI camera that is mounted above the fish tank with a frame rate 250 frames per second.
(AVI)

Video S7 **Video file showing a movie of hCLCN1^I553F/H555N-^IRES-EGFP zebrafish.** The video is recorded using the AOS™ SPRI camera that is mounted above the fish tank with a frame rate 250 frames per second.
(AVI)

Video S8 **Video file to be used for analysis using the source code provided in Information S1.** The file shows a movie of a zebrafish, recorded using the AOS™ SPRI camera that is mounted above the fish tank with a frame rate 250 frames per second.
(AVI)

Information S1 **Matlab implementation of video analytics system for zebrafish.** The system is fully described in the Material and Methods. The system is implemented in Matlab 2008b and Win7. There is no guarantee it will run on other operating systems or Matlab versions. Basic usage instructions: 1. Start Matlab 2. Load a video file (for example S8). 3. Use ‘S9.m’ to obtain various parameters of the zebrafish motion in the video.
(M) Click here for additional data file.
